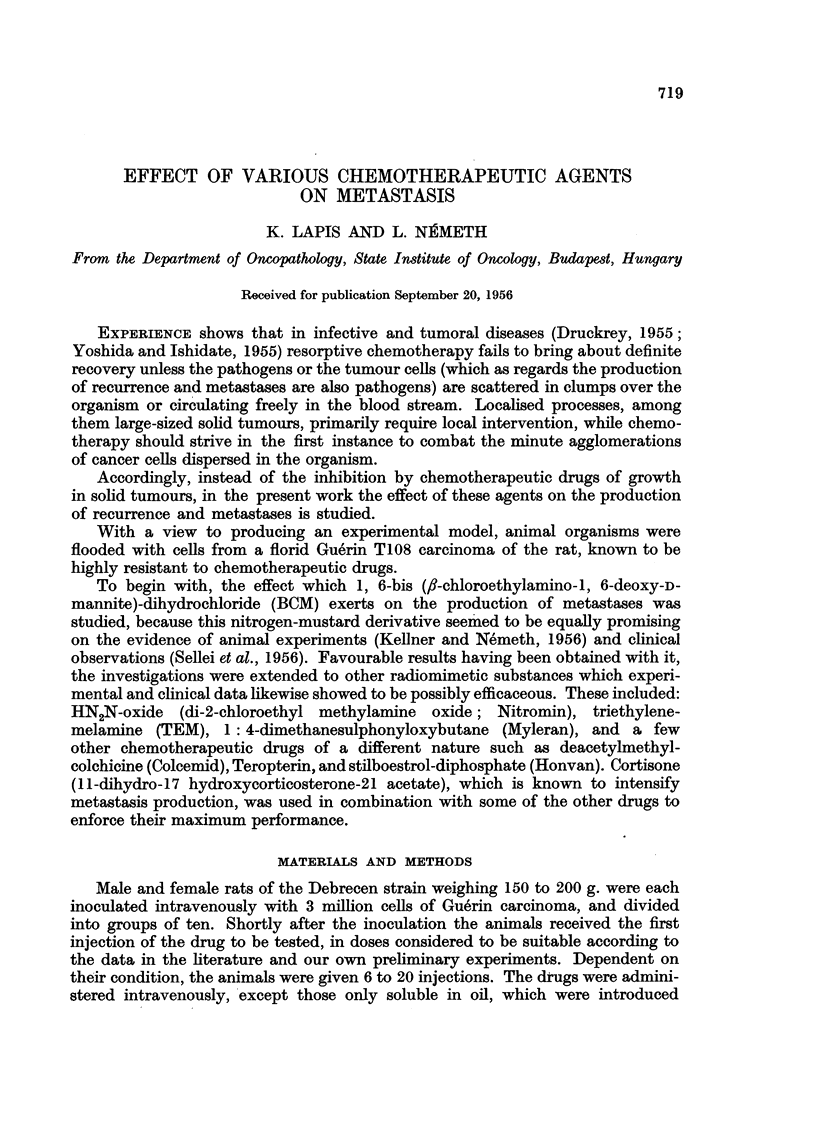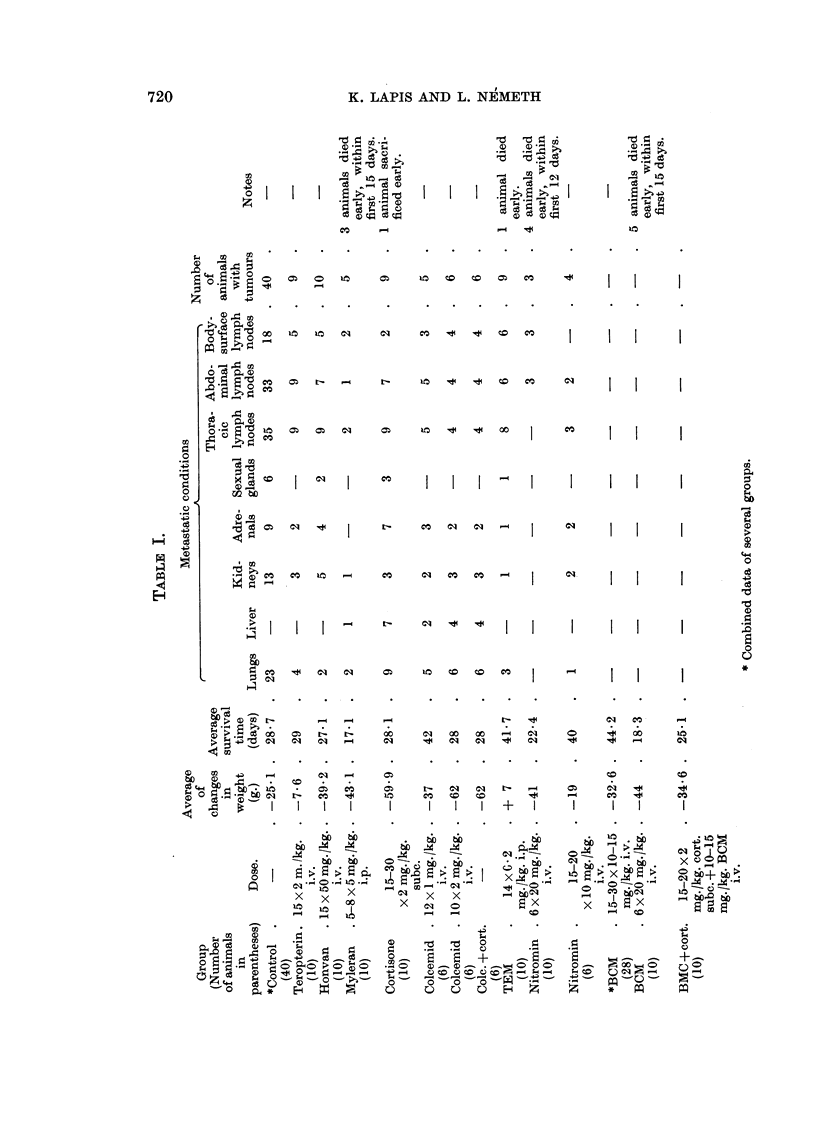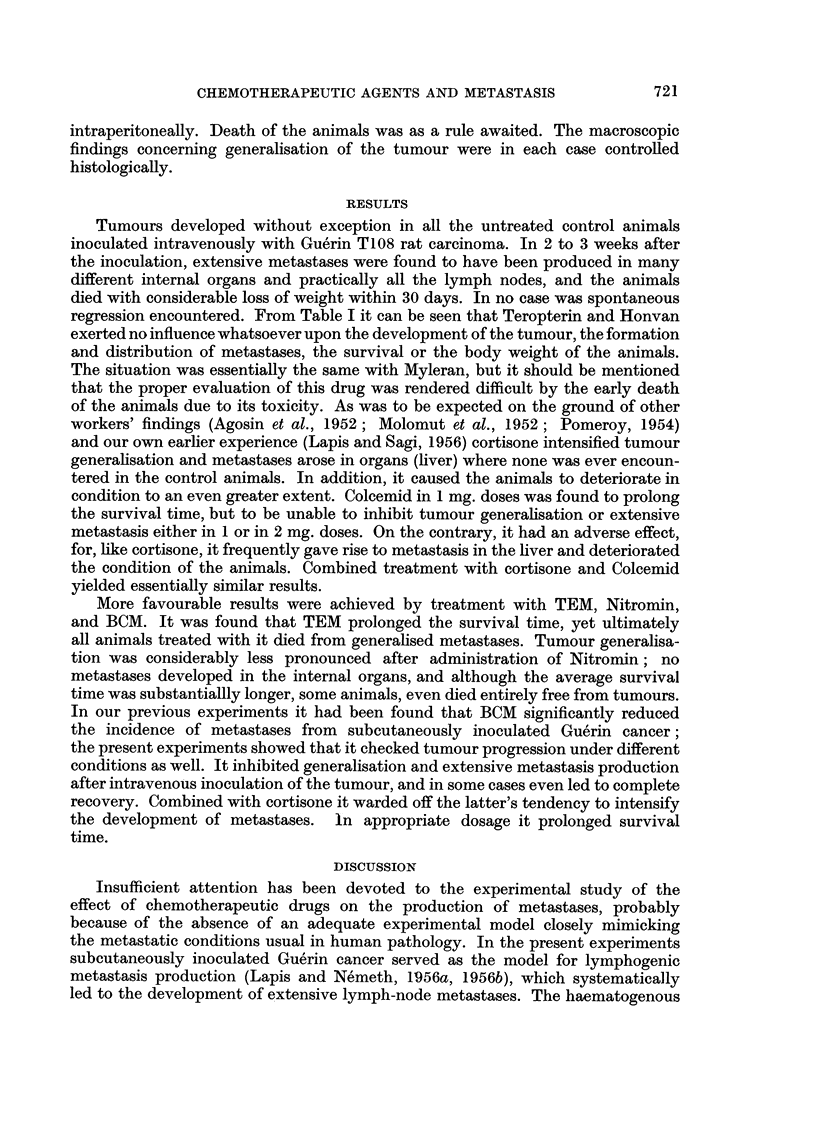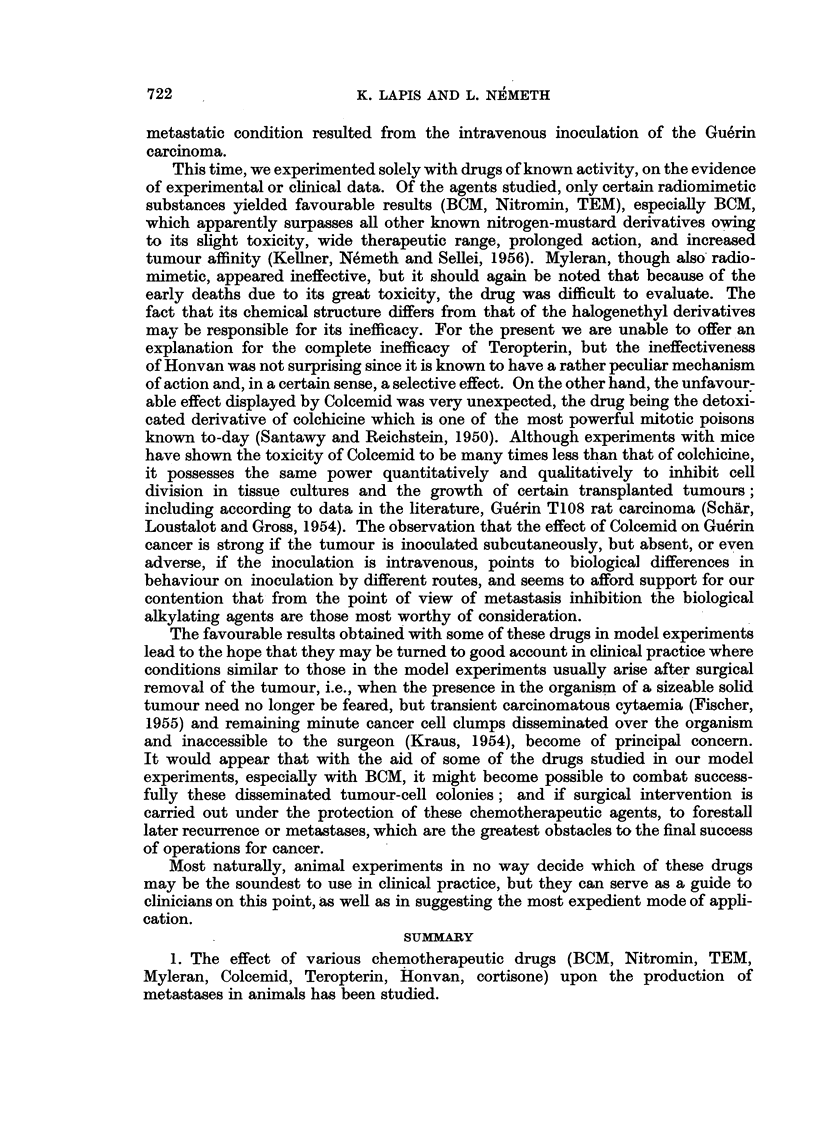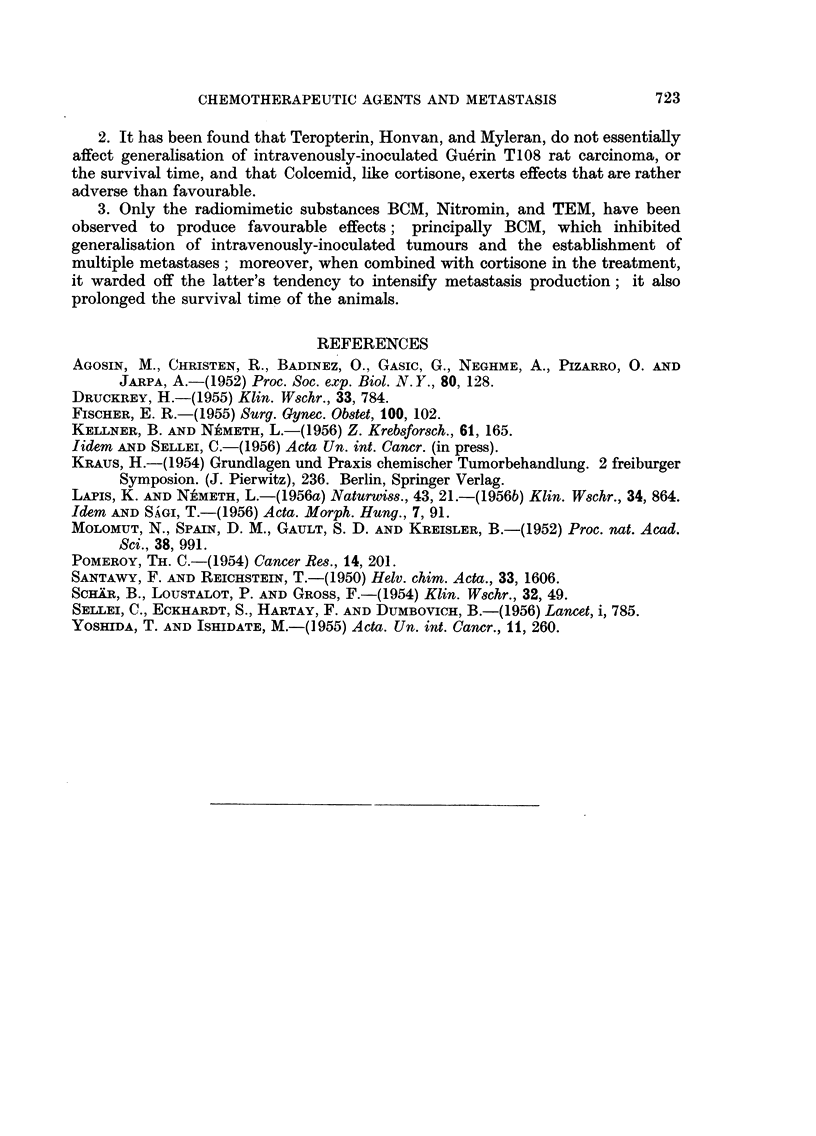# Effect of Various Chemotherapeutic Agents on Metastasis

**DOI:** 10.1038/bjc.1956.87

**Published:** 1956-12

**Authors:** K. Lapis, L. Németh


### EFFECT OF VARIOUS CHEMOTHERAPEUTIC AGENTS ON METASTASIS

K. LAPIS AND L. NÉMETH

*From the Department of Oncopathology, State Institute of Oncology, Budapest, Hungary*

Received for publication September 20, 1956

EXPERIENCE shows that in infective and tumoral diseases (Druckrey, 1955; Yoshida and Ishidate, 1955) resorptive chemotherapy fails to bring about definite recovery unless the pathogens or the tumour cells (which as regards the production of recurrence and metastases are also pathogens) are scattered in clumps over the organism or circulating freely in the blood stream. Localised processes, among them large-sized solid tumours, primarily require local intervention, while chemotherapy should strive in the first instance to combat the minute agglomerations of cancer cells dispersed in the organism.

Accordingly, instead of the inhibition by chemotherapeutic drugs of growth in solid tumours, in the present work the effect of these agents on the production of recurrence and metastases is studied.

With a view to producing an experimental model, animal organisms were flooded with cells from a florid Guérin T108 carcinoma of the rat, known to be highly resistant to chemotherapeutic drugs.

To begin with, the effect which 1, 6-bis ($\beta$-chloroethylamino-1, 6-deoxy-D-mannite)-dihydrochloride (BCM) exerts on the production of metastases was studied, because this nitrogen-mustard derivative seemed to be equally promising on the evidence of animal experiments (Kellner and Németh, 1956) and clinical observations (Sellei *et al.*, 1956). Favourable results having been obtained with it, the investigations were extended to other radiomimetic substances which experimental and clinical data likewise showed to be possibly efficaceous. These included: $HN_2N$-oxide (di-2-chloroethyl methylamine oxide; Nitromin), triethylene-melamine (TEM), 1 : 4-dimethanesulphonyloxybutane (Myleran), and a few other chemotherapeutic drugs of a different nature such as deacetylmethyl-colchicine (Colcemid), Teropterin, and stilboestrol-diphosphate (Honvan). Cortisone (11-dihydro-17 hydroxycorticosterone-21 acetate), which is known to intensify metastasis production, was used in combination with some of the other drugs to enforce their maximum performance.

### MATERIALS AND METHODS

Male and female rats of the Debrecen strain weighing 150 to 200 g. were each inoculated intravenously with 3 million cells of Guérin carcinoma, and divided into groups of ten. Shortly after the inoculation the animals received the first injection of the drug to be tested, in doses considered to be suitable according to the data in the literature and our own preliminary experiments. Dependent on their condition, the animals were given 6 to 20 injections. The drugs were administered intravenously, except those only soluble in oil, which were introduced

### TABLE I.

| Group (Number of animals in parentheses) | Dose. | Average of changes in weight (g.) | Average survival time (days) | Metastatic conditions: Lungs | Liver | Kid-neys | Adre-nals | Sexual glands | Thora-cic lymph nodes | Abdo-minal lymph nodes | Body-surface lymph nodes | Number of animals with tumours | Notes |
|---|---|---|---|---|---|---|---|---|---|---|---|---|---|
| *Control (40) | — | −25·1 | 28·7 | 23 | — | 13 | 9 | 6 | 35 | 33 | 18 | 40 | — |
| Teropterin (10) | 15 × 2 m./kg. i.v. | −7·6 | 29 | 4 | — | 3 | 2 | — | 9 | 9 | 5 | 9 | — |
| Honvan (10) | 15 × 50 mg./kg. i.v. | −39·2 | 27·1 | 2 | — | 5 | 4 | 2 | 9 | 7 | 5 | 10 | — |
| Myleran (10) | 5–8 × 5 mg./kg. i.p. | −43·1 | 17·1 | 2 | 1 | 1 | — | — | 2 | 1 | 2 | 5 | 3 animals died early, within first 15 days. |
| Cortisone (10) | 15–30 × 2 mg./kg. subc. | −59·9 | 28·1 | 9 | 7 | 3 | 7 | 3 | 9 | 7 | 2 | 9 | 1 animal sacrificed early. |
| Colcemid (6) | 12 × 1 mg./kg. i.v. | −37 | 42 | 5 | 2 | 2 | 3 | — | 5 | 5 | 3 | 5 | — |
| Colcemid (6) | 10 × 2 mg./kg. i.v. | −62 | 28 | 6 | 4 | 3 | 2 | — | 4 | 4 | 4 | 6 | — |
| Colc. + cort. (6) | — | −62 | 28 | 6 | 4 | 3 | 2 | — | 4 | 4 | 4 | 6 | — |
| TEM (10) | 14 × 0·2 mg./kg. i.p. | + 7 | 41·7 | 3 | — | 1 | 1 | 1 | 8 | 6 | 6 | 9 | 1 animal died early. |
| Nitromin (10) | 6 × 20 mg./kg. i.v. | −41 | 22·4 | — | — | — | — | — | — | 3 | 3 | 3 | 4 animals died early, within first 12 days. |
| Nitromin (6) | 15–20 × 10 mg./kg. i.v. | −19 | 40 | 1 | — | 2 | 2 | — | 3 | 2 | — | 4 | — |
| *BCM (28) | 15–30 × 10–15 mg./kg. i.v. | −32·6 | 44·2 | — | — | — | — | — | — | — | — | — | — |
| BCM (10) | 6 × 20 mg./kg. i.v. | −44 | 18·3 | — | — | — | — | — | — | — | — | — | 5 animals died early, within first 15 days. |
| BMC + cort. (10) | 15–20 × 2 mg./kg. cort. subc. + 10–15 mg./kg. BCM i.v. | −34·6 | 25·1 | — | — | — | — | — | — | — | — | — | |

* Combined data of several groups.

intraperitoneally. Death of the animals was as a rule awaited. The macroscopic findings concerning generalisation of the tumour were in each case controlled histologically.

### RESULTS

Tumours developed without exception in all the untreated control animals inoculated intravenously with Guérin T108 rat carcinoma. In 2 to 3 weeks after the inoculation, extensive metastases were found to have been produced in many different internal organs and practically all the lymph nodes, and the animals died with considerable loss of weight within 30 days. In no case was spontaneous regression encountered. From Table I it can be seen that Teropterin and Honvan exerted no influence whatsoever upon the development of the tumour, the formation and distribution of metastases, the survival or the body weight of the animals. The situation was essentially the same with Myleran, but it should be mentioned that the proper evaluation of this drug was rendered difficult by the early death of the animals due to its toxicity. As was to be expected on the ground of other workers' findings (Agosin *et al.*, 1952 ; Molomut *et al.*, 1952 ; Pomeroy, 1954) and our own earlier experience (Lapis and Sagi, 1956) cortisone intensified tumour generalisation and metastases arose in organs (liver) where none was ever encountered in the control animals. In addition, it caused the animals to deteriorate in condition to an even greater extent. Colcemid in 1 mg. doses was found to prolong the survival time, but to be unable to inhibit tumour generalisation or extensive metastasis either in 1 or in 2 mg. doses. On the contrary, it had an adverse effect, for, like cortisone, it frequently gave rise to metastasis in the liver and deteriorated the condition of the animals. Combined treatment with cortisone and Colcemid yielded essentially similar results.

More favourable results were achieved by treatment with TEM, Nitromin, and BCM. It was found that TEM prolonged the survival time, yet ultimately all animals treated with it died from generalised metastases. Tumour generalisation was considerably less pronounced after administration of Nitromin ; no metastases developed in the internal organs, and although the average survival time was substantiallly longer, some animals, even died entirely free from tumours. In our previous experiments it had been found that BCM significantly reduced the incidence of metastases from subcutaneously inoculated Guérin cancer ; the present experiments showed that it checked tumour progression under different conditions as well. It inhibited generalisation and extensive metastasis production after intravenous inoculation of the tumour, and in some cases even led to complete recovery. Combined with cortisone it warded off the latter's tendency to intensify the development of metastases. In appropriate dosage it prolonged survival time.

### DISCUSSION

Insufficient attention has been devoted to the experimental study of the effect of chemotherapeutic drugs on the production of metastases, probably because of the absence of an adequate experimental model closely mimicking the metastatic conditions usual in human pathology. In the present experiments subcutaneously inoculated Guérin cancer served as the model for lymphogenic metastasis production (Lapis and Németh, 1956*a*, 1956*b*), which systematically led to the development of extensive lymph-node metastases. The haematogenous metastatic condition resulted from the intravenous inoculation of the Guérin carcinoma.

This time, we experimented solely with drugs of known activity, on the evidence of experimental or clinical data. Of the agents studied, only certain radiomimetic substances yielded favourable results (BCM, Nitromin, TEM), especially BCM, which apparently surpasses all other known nitrogen-mustard derivatives owing to its slight toxicity, wide therapeutic range, prolonged action, and increased tumour affinity (Kellner, Németh and Sellei, 1956). Myleran, though also radiomimetic, appeared ineffective, but it should again be noted that because of the early deaths due to its great toxicity, the drug was difficult to evaluate. The fact that its chemical structure differs from that of the halogenethyl derivatives may be responsible for its inefficacy. For the present we are unable to offer an explanation for the complete inefficacy of Teropterin, but the ineffectiveness of Honvan was not surprising since it is known to have a rather peculiar mechanism of action and, in a certain sense, a selective effect. On the other hand, the unfavourable effect displayed by Colcemid was very unexpected, the drug being the detoxicated derivative of colchicine which is one of the most powerful mitotic poisons known to-day (Santawy and Reichstein, 1950). Although experiments with mice have shown the toxicity of Colcemid to be many times less than that of colchicine, it possesses the same power quantitatively and qualitatively to inhibit cell division in tissue cultures and the growth of certain transplanted tumours; including according to data in the literature, Guérin T108 rat carcinoma (Schär, Loustalot and Gross, 1954). The observation that the effect of Colcemid on Guérin cancer is strong if the tumour is inoculated subcutaneously, but absent, or even adverse, if the inoculation is intravenous, points to biological differences in behaviour on inoculation by different routes, and seems to afford support for our contention that from the point of view of metastasis inhibition the biological alkylating agents are those most worthy of consideration.

The favourable results obtained with some of these drugs in model experiments lead to the hope that they may be turned to good account in clinical practice where conditions similar to those in the model experiments usually arise after surgical removal of the tumour, i.e., when the presence in the organism of a sizeable solid tumour need no longer be feared, but transient carcinomatous cytaemia (Fischer, 1955) and remaining minute cancer cell clumps disseminated over the organism and inaccessible to the surgeon (Kraus, 1954), become of principal concern. It would appear that with the aid of some of the drugs studied in our model experiments, especially with BCM, it might become possible to combat successfully these disseminated tumour-cell colonies; and if surgical intervention is carried out under the protection of these chemotherapeutic agents, to forestall later recurrence or metastases, which are the greatest obstacles to the final success of operations for cancer.

Most naturally, animal experiments in no way decide which of these drugs may be the soundest to use in clinical practice, but they can serve as a guide to clinicians on this point, as well as in suggesting the most expedient mode of application.

### SUMMARY

1. The effect of various chemotherapeutic drugs (BCM, Nitromin, TEM, Myleran, Colcemid, Teropterin, Honvan, cortisone) upon the production of metastases in animals has been studied.

2. It has been found that Teropterin, Honvan, and Myleran, do not essentially affect generalisation of intravenously-inoculated Guérin T108 rat carcinoma, or the survival time, and that Colcemid, like cortisone, exerts effects that are rather adverse than favourable.

3. Only the radiomimetic substances BCM, Nitromin, and TEM, have been observed to produce favourable effects; principally BCM, which inhibited generalisation of intravenously-inoculated tumours and the establishment of multiple metastases; moreover, when combined with cortisone in the treatment, it warded off the latter's tendency to intensify metastasis production; it also prolonged the survival time of the animals.

### REFERENCES

Agosin, M., Christen, R., Badinez, O., Gasic, G., Neghme, A., Pizarro, O. and Jarpa, A.—(1952) *Proc. Soc. exp. Biol. N.Y.*, **80**, 128.

Druckrey, H.—(1955) *Klin. Wschr.*, **33**, 784.

Fischer, E. R.—(1955) *Surg. Gynec. Obstet*, **100**, 102.

Kellner, B. and Németh, L.—(1956) *Z. Krebsforsch.*, **61**, 165.

*Iidem* and Sellei, C.—(1956) *Acta Un. int. Cancr.* (in press).

Kraus, H.—(1954) Grundlagen und Praxis chemischer Tumorbehandlung. 2 freiburger Symposion. (J. Pierwitz), 236. Berlin, Springer Verlag.

Lapis, K. and Németh, L.—(1956*a*) *Naturwiss.*, 43, 21.—(1956*b*) *Klin. Wschr.*, **34**, 864.

*Idem* and Sági, T.—(1956) *Acta. Morph. Hung.*, **7**, 91.

Molomut, N., Spain, D. M., Gault, S. D. and Kreisler, B.—(1952) *Proc. nat. Acad. Sci.*, **38**, 991.

Pomeroy, Th. C.—(1954) *Cancer Res.*, **14**, 201.

Santawy, F. and Reichstein, T.—(1950) *Helv. chim. Acta.*, **33**, 1606.

Schär, B., Loustalot, P. and Gross, F.—(1954) *Klin. Wschr.*, **32**, 49.

Sellei, C., Eckhardt, S., Hartay, F. and Dumbovich, B.—(1956) *Lancet*, i, 785.

Yoshida, T. and Ishidate, M.—(1955) *Acta. Un. int. Cancr.*, **11**, 260.